# Does educational attainment modify the causal relationship between adiposity and cardiovascular disease? A Mendelian randomization study

**DOI:** 10.1016/j.ssmph.2023.101351

**Published:** 2023-01-30

**Authors:** Robert C. Schell, William H. Dow, Lia C.H. Fernald, Patrick T. Bradshaw, David H. Rehkopf

**Affiliations:** a Division of Health Policy and Management, School of Public Health, University of California, Berkeley, USA; b Department of Demography, University of California, Berkeley, CA, USA; c Division of Community Health Sciences, School of Public Health, University of California, Berkeley, CA, USA; d Division of Epidemiology & Biostatistics, University of California, School of Public Health, Berkeley, Berkeley, CA, USA; e Department of Epidemiology and Population Health, Stanford University, Palo Alto, CA, USA

**Keywords:** Body mass index, Mendelian randomization, Adiposity, Educational attainment, UK Biobank, Cardiovascular disease, Causal inference

## Abstract

A greater risk of cardiovascular disease is associated with low educational attainment and high adiposity. Despite the correlation between low educational attainment and high adiposity, whether educational attainment modifies the risk of CVD caused by high adiposity remains poorly understood. We investigated the effect of adiposity (body mass index [BMI] and waist-to-hip ratio adjusted for BMI [WHRadjBMI]) on incident CVD among individuals with varying education levels, using associational and one-sample Mendelian randomization (MR) survival analyses. Data were collected from 2006 to 2021, and sample sizes were 254,281 (27,511 CVD cases) for BMI and 253,968 (27,458 CVD cases) for WHRadjBMI. In the associational model, a standard deviation (SD) higher BMI was associated with 19.81 (95% CI: 18.55–21.06) additional cases of incident CVD per 10,000 person-years for individuals with a secondary education, versus 32.96 (95% CI: 28.75–37.17) for those without. When university degree served as the education variable, education group differences attenuated, with 18.26 (95% CI: 16.37–20.15) cases from a one SD higher BMI for those with a university degree versus 23.18 [95% CI: 21.56–24.72] for those without. For the MR model, an SD higher BMI resulted in 11.75 (95% CI: −0.84-24.38) and 29.79 (95% CI: 17.20–42.44) additional cases of incident CVD per 10,000 person-years for individuals with versus without a university degree. WHRadjBMI exhibited no effect differences by education. While the associational model showed evidence of educational attainment modifying the relationship between adiposity and incident CVD, it does not modify the association between adiposity and incident CVD in the MR models. This suggests either less education does not cause greater risk of incident CVD from high adiposity, or MR models cannot detect the effect difference. The associational point estimates exist within the MR models’ confidence intervals in all BMI analyses, so we cannot rule out the effect sizes in the associational models.

## Highlights

• This study is among the first to treat education as potential modifier for adiposity's effect on cardiovascular disease (CVD).
• People with different levels of educational attainment faced different hazards of CVD from increased adiposity in associational models.
• Only individuals with a university degree and their peers with less education experienced different hazards in the MR models.
• Educational attainment does not appear to significantly modify the association between adiposity and incident CVD.

## 1 Introduction

One of the great public health achievements ever has been the substantial reduction in cardiovascular disease (CVD) incidence and mortality in high-income countries (Koppaka, 2011; Murray et al., 2012; Tran et al., 2018). However, these reductions were not equally distributed, and people from lower socioeconomic strata, for example as defined by lower educational attainment, and people with higher levels of adiposity still face a disproportionately high risk of experiencing a CVD event (Cao & Cui, 2020; Carter et al., 2019a; Emdin et al., 2017; Khaing et al., 2017; Loucks et al., 2014; Winkleby et al., 1992). A recent meta-analysis of observational studies found that people with a high school education or less faced a 27%–50% greater risk of a CVD event, while controlling for body mass index (BMI) (Khaing et al., 2017). Just as low educational attainment is related to a substantial increased risk of CVD, so too is high adiposity. A recent Mendelian randomization (MR) analysis showed that a standard deviation higher waist-to-hip ratio adjusted for BMI (WHRadjBMI) is associated with an odds ratio of 1.46 for coronary heart disease (CHD), which reinforces decades of evidence from observational studies (Emdin et al., 2017; Manson et al., 1990; Yusuf et al., 2005).

These disparities in CVD risk are especially concerning given the high prevalence of obesity, at 28% in the UK, and the fact that over half of UK residents never graduate college and 21% do not have a secondary education (Baker, 2022; International, 2020; Labour Market). Educational attainment and high adiposity also tend to coexist, with one study finding that 22.5% of Europeans with only primary school education had obesity compared to 9.9% of individuals with a university degree (Hermann et al., 2011). Despite their high prevalence and tendency to co-occur, the degree to which low educational attainment modifies the risk of CVD caused by high adiposity remains poorly understood.

MR is an instrumental variable technique that relies on random genetic variation as a natural experiment and, if the core assumptions are met, provides a causal effect robust to reverse causation and confounding bias. The elimination of reverse causation is especially important when considering adiposity as an exposure because of its tendency to blur the association of illness and adiposity in older age (Flegal et al., 2011). While a person's adiposity derives from both genetic and lifestyle factors, MR provides a unique opportunity to isolate the health effects of adiposity from other confounding health behaviors, such as smoking, that could affect CVD risk and adiposity. Recent MR studies on adiposity's effect on CVD improve on the methodological limitations of the earliest studies, but they still largely treat educational attainment as a confounder for which to adjust (Farmer et al., 2019; Hägg et al., 2015; Larsson et al., 2020; Riaz et al., 2018). However, two people with identical levels of adiposity and different levels of education may face different associations between adiposity and CVD risk. Differences in incident CVD risk from adiposity could exist between educational attainment groups because of differences in medical care access and utilization, alcohol consumption patterns, and from higher levels of inflammation, hypertension, and hyperlipidemia (Beauchamp et al., 2010; Carter et al., 2019b; Dégano et al., 2017; Gill et al., 2021; Havranek et al., 2015; Loucks et al., 2014; Schultz et al., 2018; Winkleby et al., 1992). This study is the first to directly explore how different levels of education modify the effect of adiposity on incident CVD.

**Fig. 1:**
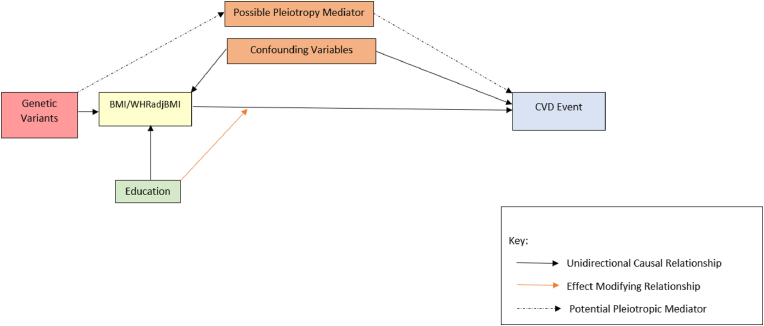
Directed acyclic graph of adiposity, educational attainment, and cardiovascular disease incidence.

We estimate the relationship between adiposity and incident CVD at different levels of education via an MR survival analysis. We hypothesize that an increase in adiposity leads to a greater incidence of CVD among adults without a secondary education in the UK Biobank (UKB) compared to the risk faced by their better educated peers, as shown in the DAG in Fig. 1. We also explore potential effect heterogeneity for individuals with more or less than a university degree.

## 2 Methods

**Fig. 2:**
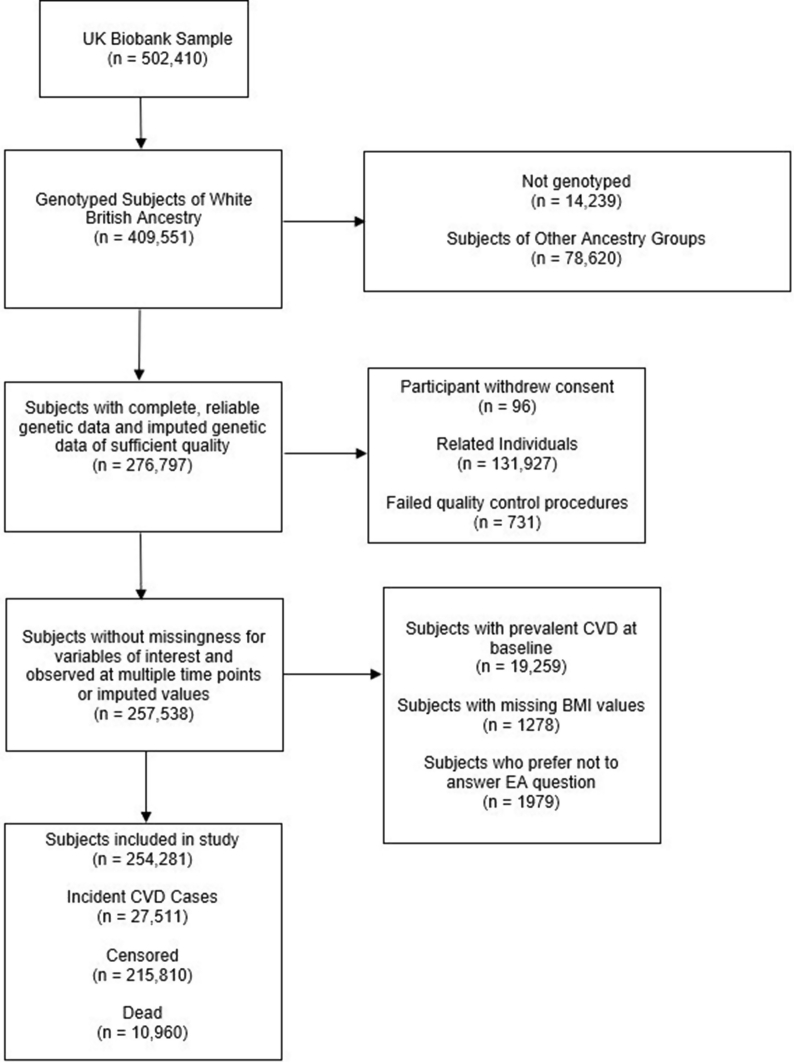
Subject exclusion flowchart.

The UKB is a population-based cohort of over 500,000 people from England, Scotland, and Wales aged 40–69 years old at recruitment designed to explore the genetic and environmental determinants of a variety of diseases (Sudlow et al., 2015). It began in 2006 and is uniquely suited to perform a One Sample MR survival analysis because of its size and the wide array of data collected longitudinally (Bycroft et al., 2018). We included only a subset of the full UKB dataset based on the criteria outlined in Fig. 2 and discussed in detail in the supplement. MR requires us to restrict to unrelated individuals with high-quality genetic data. Because of the possibility of population stratification, or spurious associations which can occur if a disease and a genetic variant are more or less common in a specific ancestry group, we must also restrict to only individuals of white British ancestry. Pooled analysis of ancestry groups would create these spurious associations and there are simply too few non-white subjects in the UK Biobank to produce separate, well-powered analyses in these other ancestry groups.

### 2.1 Incident cardiovascular disease case definition

The main outcome of interest in this study is time to incident cardiovascular disease (CVD), defined as angina, myocardial infarction, ischemic heart disease, heart failure, and stroke with the following ICD-10 codes (and OPCS-4 codes for operations): I20 to I25, I44, I50, I60 to I64, I69, K40 to K46, K49, K50, K75 (World Health Organization, 2004, pp. 1–677; Connecting for Health (Organization), 2009). If ICD-10 codes were unavailable for an individual, we relied on the corresponding self-reported events. We focused on incident instead of prevalent CVD, so only CVD cases that occurred after the first adiposity measurement for a subject who has not yet experienced CVD qualified as events.

#### 2.1.1 Educational attainment

We treated education as a self-reported categorical variable separated into individuals who did not complete secondary education and those that did. In secondary analyses, we also split education into those with and without a university degree. We chose these dichotomizations of educational attainment because past studies have shown significant differences in CVD risk between these educational groups (Khaing et al., 2017; Loucks et al., 2014; Winkleby et al., 1992).

### 2.2 Adiposity

We used first-measured WHRadjBMI, defined as the ratio of the circumference of the waist compared to the hip controlling for BMI and first measured BMI as the two measures of adiposity in this study. This first adiposity measurement occurred at enrollment for all of the individuals in the present study. The benefit of using WHRadjBMI is that it has a more direct correlation with adiposity and measures central adiposity, which serves as an independent predictor of CVD risk beyond total body adiposity (Prentice & Jebb, 2001) (Dale et al., 2017). We stratified the WHRadjBMI analysis by sex due to the sexually dimorphic nature of the exposure (Shungin et al., 2015). Therefore, the BMI models are presented pooled by biological sex, while the WHRadjBMI models are presented stratified by sex. Both exposures were standardized by subtracting the mean from each individual value and dividing by the standard deviation.

### 2.3 Selected genetic variants

We identified 97 variants significantly associated with BMI that explain 2.7% of its variance from the most recent genome-wide association study (GWAS) that excluded the UKB (Locke et al., 2015). We selected the 49 variants associated with an elevated WHRadjBMI at genome-wide significance based on a recent GWAS performed by the GIANT Consortium (Shungin et al., 2015). The supplement provides a detailed description of genetic variant quality control and imputation. We performed additional confirmatory analyses as reported in Supplement Tables S2 and S3 to ensure the genetic data's accuracy by comparing our genetic effect estimates to those in the original GWAS and those produced by the Neale Lab, a lab that focuses on replicating GWAS results in the UKB (Neale Lab, 2018; Locke et al., 2015; Shungin et al., 2015).

These genetic variants can serve as valid instrumental variables for adiposity subject to three assumptions: relevance, exchangeability, and no horizontal pleiotropy (Sheehan et al., 2008). Relevance, which requires that the variant influences adiposity, represents the only empirically verifiable assumption. We verify relevance by determining whether the instruments combine to have a partial F-statistic over 10, a conventional threshold for instrument strength. Exchangeability, or a lack of confounding between the outcome and variant, seems plausible in this scenario given the random assignment of genes at birth, although population stratification could violate it. The last assumption, no horizontal pleiotropy, is the most contentious and likely to fail in practice. This happens when a variant affects CVD risk both through adiposity and some other exposure.

### 2.4 Statistical analyses

The following analyses are stratified by educational attainment, with subjects grouped as those with versus without a secondary education and those with and without a university degree. All analyses consist of an associational model and an MR model. We used Aalen's additive hazards model for both the MR and associational survival analyses. We chose the additive hazard model instead of the Cox model to avoid the issue of non-collapsibility of the hazard ratio as a measure of association (Cho et al., 2021; Martinussen & Vansteelandt, 2013). In a survival model with death as a competing risk, the quantity estimated is a cause-specific hazard difference. The timescale used is time since first measured adiposity. Time to event, the outcome of interest, signifies time from first measurement of adiposity to first incident CVD event. We controlled for baseline age, genotyping array, whether a subject ever smoked (defined as over 100 lifetime cigarettes), the first ten genetic ancestry principal components as a standard way to further control for population stratification confounding, and biological sex in the BMI models (the WHRadjBMI models stratify by sex). We relied on complete case analysis due to low rates of missingness in the UKB with only 3257 individuals missing exposure or outcome values at the final stage of screening.

For the MR analyses we first performed inverse variance weighted (IVW) regression, which is the most efficient MR estimator because it more heavily weights variants with more precise effects on adiposity and, thus, CVD incidence (Lee et al., 2016). We utilized a fixed effects modeling approach, which assumes one underlying “true” effect of adiposity on CVD incidence in each educational attainment stratum. While the IVW regression provides the most statistical efficiency of any MR design, it is also the most susceptible to bias due to horizontal pleiotropy (Bowden et al., 2016).

We performed sensitivity analyses that vary the assumptions underlying the model to make the causal effect more plausible. The weighted median estimator is an alternative model utilized in many MR analyses that offers less efficiency than the IVW estimator but provides robustness to certain forms of horizontal pleiotropy (Bowden et al., 2016). The weighted median estimator is consistent provided at least half of its weight is placed on variants acting as valid instruments. The causal effect is then the effect of the variant at the 50^th^ percentile of the weights (Bowden et al., 2016). Supplemental sensitivity analyses are in Figs. S4–S6. Because the MR estimators do not allow formal assessments of effect modification, we performed stratified analyses and evaluated confidence intervals and point estimates. While non-overlapping confidence intervals imply statistically significant differences, their overlap does not imply that no difference exists and so we only interpreted confidence intervals in the cases where they diverged or overlapped extensively, thus our conclusions should be conservative (Payton et al., 2003). All analyses were performed using R 4.1.3, with code and steps to replicate this analysis available at https://github.com/BobbySchell. Because individuals with at least (less than) a secondary education and with (without) a university degree overlap, we focused on comparisons between mutually exclusive groups. We pre-specified all analyses and hypotheses on Open Science Framework at https://github.com/BobbySchell. This study follows the STROBE-MR guidelines, available in the supplement as Table S1 (Smith et al., 2019).

## 3 Results

Out of the 254,281 (253,968) subjects that fit the inclusion criteria for the BMI (WHRadjBMI) analysis, 27,511 (27,458) experienced a CVD incident over 2,970,344 (2,969,741) person-years. Subjects with more education had lower average BMIs and incidence of CVD, were less likely to smoke, and were generally younger at baseline than their peers with less education (Table 1, Table 2). The genetic variants displayed relationships of adequate strength according to our pre-analysis plan for every group except for subjects with less than secondary education and males with at least a university degree for WHRadjBMI, as the partial F-statistic for each of the rest exceeded 10. It is important to note that this instrument strength estimate was derived from estimating a first-stage regression with the genetic variants as variables and the confounders, which differs from the models used in the results. Figs. S1–S3 present a detailed breakdown of follow-up time by event, Kaplan-Meier curves for overall survival probability, and cumulative incidence by event type.

**Table 1:** Characteristics of cohorts with less than and at least secondary education.

	Less than Secondary Education		At Least Secondary Education	
	Mean (SD)	Range	Mean (SD)	Range
Age at Baseline	61.55 (6.28)	40.31–71.19	56.16 (7.97)	40.02–72.95
BMI at Baseline	28.17 (4.84)	14.28–68.41	27.05 (4.65)	12.12–74.68
WHR at Baseline^a^	0.89 (0.09)	0.54–1.56	0.86 (0.09)	0.20–1.65
Follow Up Time	11.23 (3.05)	0.005–14.45	11.78 (2.41)	0.0027–15.55
CVD Incidence	0.17 (0.38)	0–1	0.10 (0.30)	0–1
Ever Smoked	0.64 (0.48)	0–1	0.59 (0.49)	0–1
**Biological Sex**	**N (%)**		**N (%)**	
Female	21,581 (56.06%)		117,910 (54.64%)	
Male	16,915 (43.94%)		97,875 (45.36%)	
**Region**	**N (%)**		**N (%)**	
England	33,574 (87.21%)		190,418 (88.24%)	
Scotlan	3276 (8.51%)		15,814 (7.33%)	
Wales	1646 (4.28%)		9553 (4.43%)	
**Total Participants**	38,496 (100%)		215,785 (100%)	

a Note: 253,968 subjects with valid WHR measure (38,484 less than secondary education; 215,484 at least secondary education).

**Table 2:** Characteristics of cohorts with less than university degree and at least university degree.

	Less than University		At Least University	
	Mean (SD)	Range	Mean (SD)	Range
Age at Baseline	57.64 (7.94)	40.20–72.95	55.70 (7.90)	40.02–70.49
BMI at Baseline	27.63 (4.79)	12.12–74.68	26.43 (4.40)	13.12–65.23
WHR at Baseline^a^	0.87 (0.09)	0.20–1.65	0.86 (0.09)	0.45–1.48
Follow Up Time	11.60 (2.63)	0.003–15.55	11.88 (2.29)	0.005–14.46
CVD Incidence	0.12 (0.33)	0–1	0.08 (0.28)	0–1
Ever Smoked	0.61 (0.49)	0–1	0.58 (0.49)	0–1
**Biological Sex**	**N (%)**		**N (%)**	
Female	94,368 (56.21%)		45,123 (52.23%)	
Male	73,525 (43.79%)		41,265 (47.77%)	
**Region**	**N (%)**		**N (%)**	
England	149,201 (88.87%)		74,791 (86.58%)	
Scotland	11,244 (6.70%)		7846 (9.08%)	
Wales	7448 (4.44%)		3751 (4.34%)	
**Total Participants**	167,893 (100%)		86,388 (100%)	

a Note: 253,968 patients with valid WHR measure (167,595 less than university degree; 86,373 at least university degree).

**Fig. 3:**
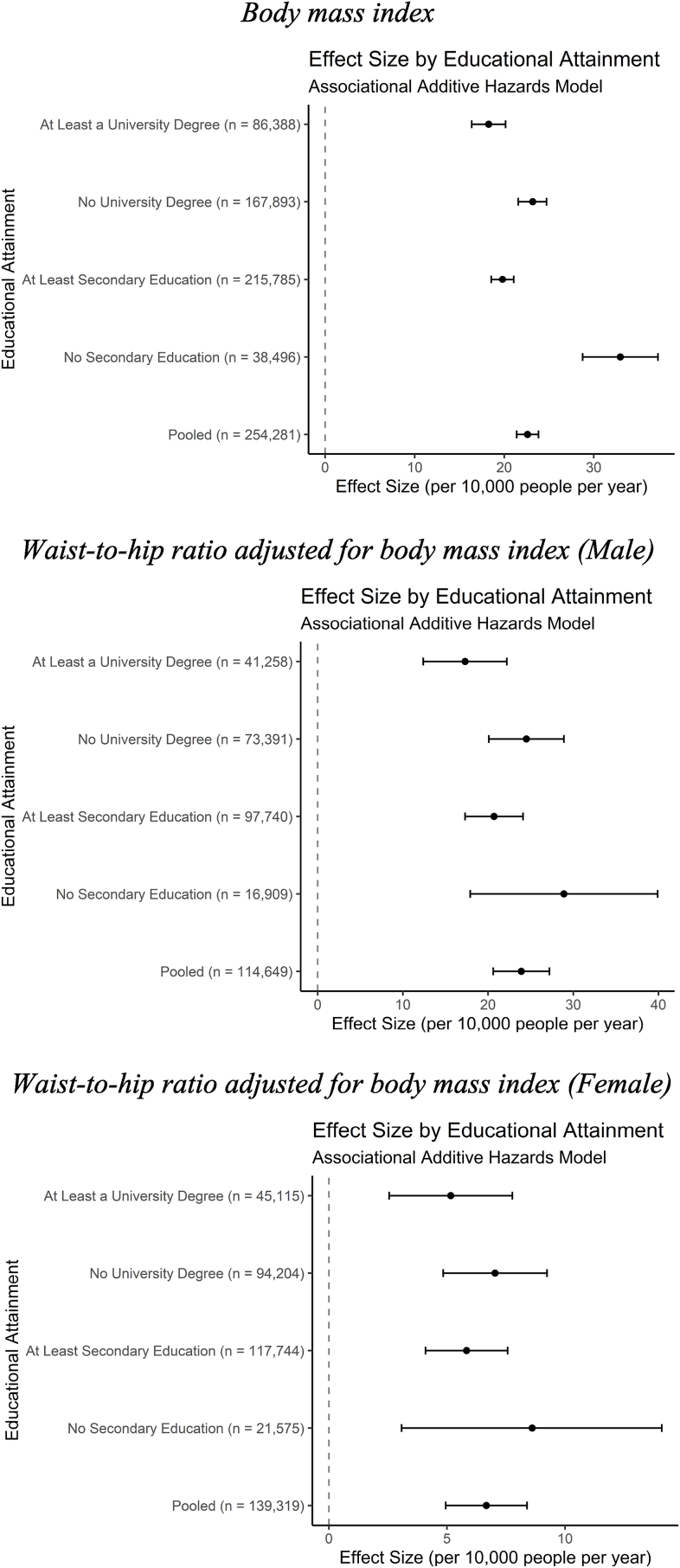
Associational relationship between adiposity and incident cardiovascular disease.

In the adjusted model in Table 3 and Fig. 3, a standard deviation higher BMI (4.69 kg/m^2^) results in 22.60 (95% CI: 21.39 to 23.82) excess cases of incident CVD per year among 10,000 individuals in the pooled sample. This effect increases to 32.96 (95% CI: 28.75 to 37.17) for individuals with less than a secondary education compared to only 19.81 (95% CI: 18.55 to 21.06) for individuals with at least a secondary education. Given the over 33% increased rate of incident CVD per standard deviation increase in BMI between individuals in these groups and their non-overlapping confidence intervals, it appears education may act as an effect modifier in the associational models. Individuals with a university degree and those without one also diverge in terms of hazard of CVD, but the effect size difference is far smaller in magnitude. These differences do not appear to exist for WHRadjBMI for either sex, as the educational groups’ effect sizes largely overlap.

**Table 3:** Association between adiposity and incident cardiovascular disease by educational status and model choice.

		Body Mass Index		WHRadjBMI (Male)		WHRadjBMI (Female)	
		Additive Hazard (CVD incidents per 10,000 person-years)	95% Confidence Interval	Additive Hazard (CVD incidents per 10,000 person-years)	95% Confidence Interval	Additive Hazard (CVD incidents per 10,000 person-years)	95% Confidence Interval
Less than Secondary Education	Associational	32.96	(28.75, 37.17)	28.90	(17.90, 39.90)	8.61	(3.08, 14.10)
	IVW	28.75	(-1.89, 59.44)	−28.37	(-64.16, 7.41)	−31.12	(-60.38,−1.87)
	Weighted Median	14.28	(-27.30, 55.85)	−28.67	(-82.10, 24.75)	−26.67	(-69.95, 16.61)
At Least Secondary Education	Associational	19.81	(18.55, 21.06)	20.70	(17.30, 24.10)	5.83	(4.09, 7.57)
	IVW	23.76	(14.23, 33.34)	25.02	(-14.44, 64.47)	11.21	(2.21, 20.20)
	Weighted Median	21.16	(6.70, 35.62)	6.26	(-46.88, 59.41)	17.39	(4.04, 30.74)
Less than University Degree	Associational	23.18	(21.56, 24.72)	24.50	(20.10, 28.90)	7.04	(4.84, 9.24)
	IVW	29.79	(17.20, 42.44)	0.34	(-56.59, 55.27)	2.29	(-8.98, 13.56)
	Weighted Median	19.40	(2.92, 35.93)	−19.87	(-88.56, 48.83)	1.03	(-16.15, 18.22)
At Least University Degree	Associational	18.26	(16.37, 20.15)	17.30	(12.40, 22.20)	5.16	(2.55, 7.77)
	IVW	11.75	(-0.84, 24.38)	38.46	(-14.66, 91.55)	7.98	(-5.81, 21.77)
	Weighted Median	12.45	(-6.86, 31.77)	34.28	(-42.43, 111.00)	8.02	(-12.35, 28.38)

**Fig. 4:**
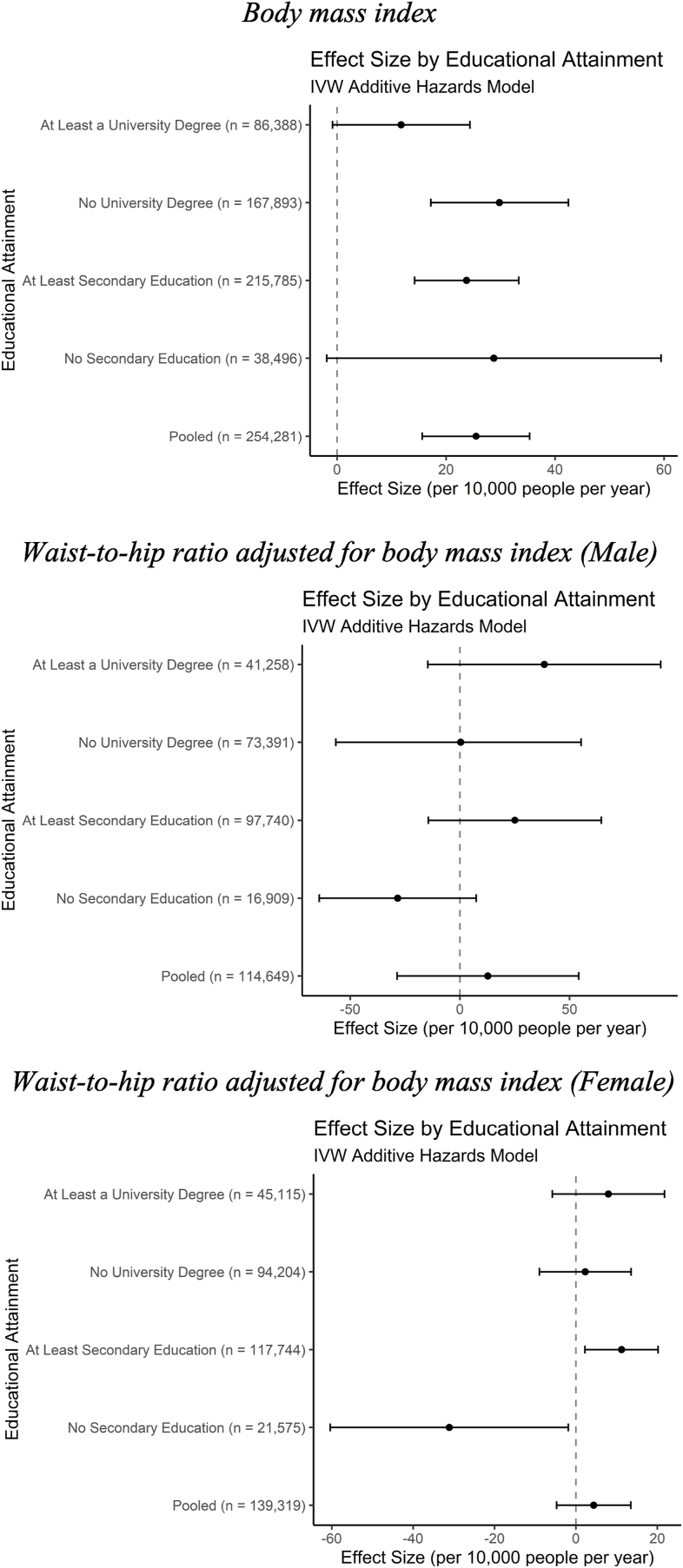
Inverse-variance weighted association between adiposity and incident cardiovascular disease.

**Fig. 5:**
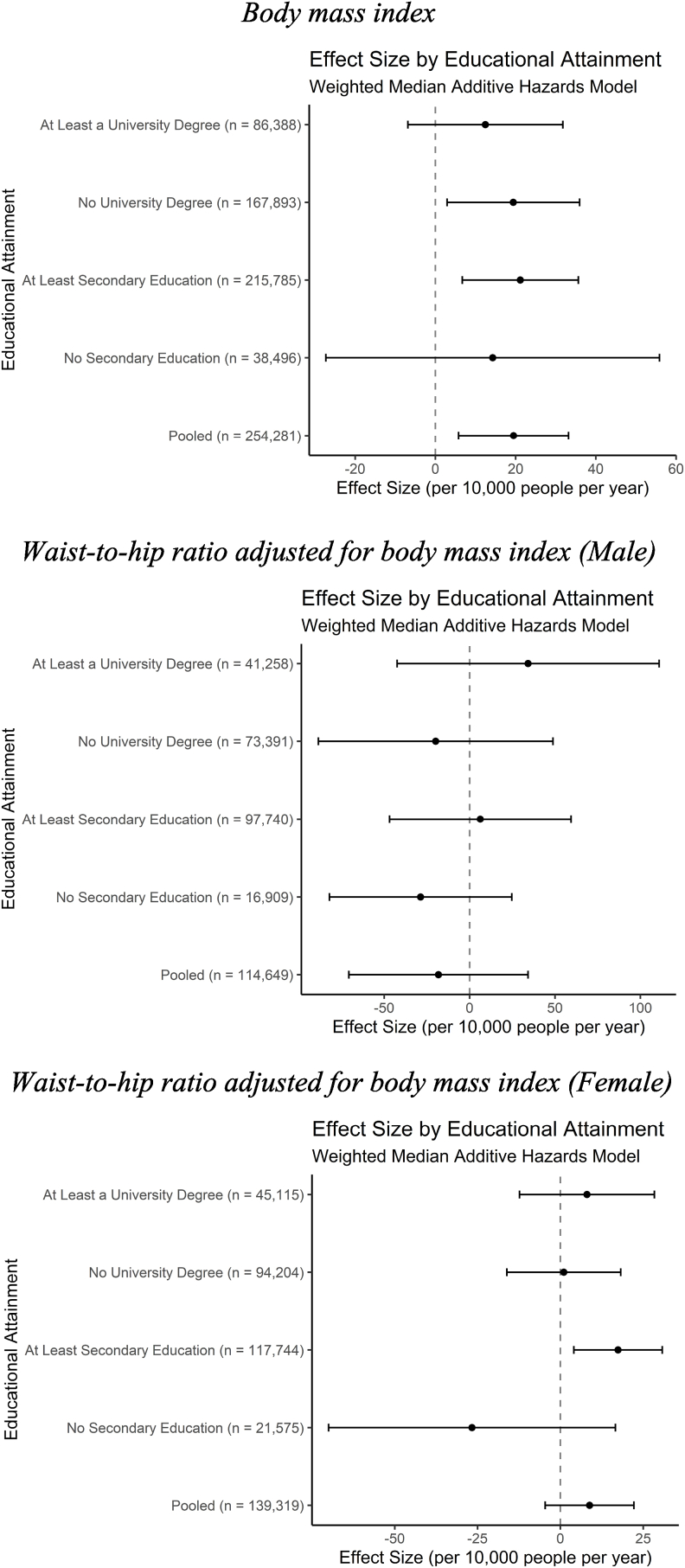
Weighted median estimator association between adiposity and incident cardiovascular disease.

In the IVW model results, shown in Fig. 4 and Table 3, the differences between educational groups for BMI appear less pronounced. For individuals with less than a secondary education, the relatively small sample size results in imprecise estimates and a confidence interval that reaches below the lowest and above the highest point of any other educational group. The point estimate for individuals with a university degree is less than half of the next closest point estimate and appears to differ meaningfully from individuals with less than a university degree. A standard deviation higher BMI results in 29.79 (95% CI: 17.20 to 42.44) excess cases of incident CVD per year among 10,000 individuals in the sample of individuals with less than a university degree compared to only 11.75 (95% CI: −0.84 to 24.38) for individuals with a university degree. Fig. 5 and Table 3 suggest that the weighted median estimator is too imprecise to draw firm conclusions about differences in hazard between educational groups.

The IVW model results for WHRadjBMI differed from our hypothesis. While the results were difficult to distinguish for males, with all groups’ confidence intervals overlapping with zero, females experienced a *decrease* in the hazard of incident CVD from a standard deviation higher WHRadjBMI. Specifically, the model suggests that a standard deviation higher WHRadjBMI results in a decrease in incidence of CVD of 31.12 per 10,000 individuals. Because some of the estimates are in opposite directions, it is important to note that the pooled estimates in this analysis have no straightforward interpretation. Likewise, in the weighted median model females experience an increase in incident CVD from a higher WHRadjBMI only if they have at least a secondary education, which also contradicts our hypothesis of an inverse association between CVD hazard and educational attainment. We repeat these analyses for household income, another dimension of socioeconomic status associated with incident CVD in the literature, in Supplement Table S2 and Figs. S7–S9 (Odutayo et al., 2017).

## 4 Discussion

The associational models in this analysis suggest differences in hazard of incident CVD from a higher BMI exist between individuals with and without a secondary education, as well as between those with and without a university degree. However, in the IVW models only individuals with and without a university degree still faced significantly different hazards of incident CVD from a higher BMI and the weighted median models were too imprecise to detect differences between educational attainment groups. Overall, we conclude that differences in hazard of incident CVD from higher BMI by educational attainment – if they exist - are small in magnitude. The results for WHRadjBMI were generally even less different between educational attainment groups, although a counterintuitive relationship emerged in the MR models for females where individuals with less than a secondary education experienced a decrease in hazard from increasing WHRadjBMI.

While higher BMI is related to an increased hazard of incident CVD for every educational attainment group in the associational and IVW models, WHRadjBMI increases the hazard of incident CVD for every group in the associational models and then only a subset of educational attainment groups thereafter. WHRadjBMI has been criticized in previous work for introducing collider bias, which could explain both the inconsistent association between WHRadjBMI and incident CVD in the MR models and the negative association found between WHRadjBMI and incident CVD in individuals with less than a secondary education (Hartwig et al., 2021).

Unlike much of the MR literature on CVD, we focused on incident CVD which should assuage concerns regarding reverse causation in studies of prevalent CVD and adiposity (Sattar & Preiss, 2017). We employed an additive hazard model instead of the more commonly used Cox model, which avoids the issue of non-collapsibility and puts the effect measure in a more easily interpreted additive form. This is the first study to our knowledge to consider SES as a potential effect modifier instead of simply as a confounder for the impact of adiposity on CVD, which acknowledges the ways in which education and adiposity could interact in the production of population health. Lastly, we utilized a variety of sensitivity analyses, perform numerous confirmatory analyses, and pre-specified the analysis plan to increase confidence in our results.

As with many MR studies, we face some statistical power limitations, especially for the smaller educational attainment subgroups. The measures of adiposity used, BMI and WHRadjBMI, have also been criticized for not capturing the underlying variable of interest (total adiposity) and producing collider bias, respectively. As with any MR analysis, we cannot ensure the assumptions outside of relevance are met or know the ‘best’ model in terms of balance between robustness and statistical efficiency. We also treated education as exogenous, as the MR design only applies to adiposity. The UK has universal healthcare and the UKB is an unusually healthy and high-status snapshot of the country (Fry et al., 2017). While health disparities by educational attainment persist in countries with universal healthcare, our analysis likely understates the differences in CVD incidence between educational attainment groups in countries without universal healthcare and in more socioeconomically diverse samples (Wang et al., 2014). Additionally, some researchers have attempted to make the UKB more representative of the broader population with sampling weights, which we do not apply in this analysis (Van Alten et al., 2022)**.** We restricted the analysis to only White Europeans and control for genetic ancestry principal components to reduce the possibility of population stratification, but social stratification remains a potential concern (Abdellaoui et al., 2019). Past evidence also suggests nonlinear effects of adiposity on CVD risk, with individuals at higher levels of adiposity facing greater risks, but we treated adiposity's impact as linear in this analysis.

Lastly, unique forms of selection bias represent a general limitation of MR studies. Because high adiposity has an association with mortality in young adulthood, the MR analysis here necessarily restricts to individuals who survived long enough to participate (Seidell, 2010; Swanson, 2019). Therefore, even with valid instruments or methods robust to invalid instruments, the possibility remains for selection bias from loss to follow up correlated with the instruments (Swanson, 2019).

## 5 Conclusion

In the associational models, individuals with lower educational attainment face a higher hazard of CVD from an increase in adiposity. However, hazard differences between educational attainment groups are only detected in the MR models for individuals with and without a university degree for BMI and not detected at all for WHRadjBMI.

## Ethical statement

UK Biobank has approval from the North West Multi-Centre Research Ethics Committee (MREC) to obtain and disseminate data and samples from the participants (http://www.ukbiobank.ac.uk/ethics/), which covers the analyses in this study. Written informed consent was obtained from all of the participants.

## Financial disclosure statement

This work was supported by 10.13039/100000002National Institutes of Health [T32-AG000246] and the 10.13039/100005595University of California Dissertation Year Fellowship. The funders had no role in study design, data collection and analysis, decision to publish, or preparation of the manuscript.

## CRediT authorship contribution statement

**Robert C. Schell:** Data curation, Conceptualization, Methodology, Writing – original draft, and, Writing – review & editing. **William H. Dow:** Data curation, Writing – review & editing. **Lia C.H. Fernald:** Data curation, Writing – review & editing. **Patrick T. Bradshaw:** Writing – review & editing. **David H. Rehkopf:** Writing – review & editing.

## Declaration of competing interest

None declared.

## Data Availability

Code and a detailed explanation of analyses performed is available at https://github.com/BobbySchell. The data must be applied for through the UK Biobank AMS.
