# Supplementary material for: Does educational attainment modify the causal relationship between adiposity and cardiovascular disease? A Mendelian randomization study
Source: SSM Popul Health. 2023 Jan 30;21:101351. doi: 10.1016/j.ssmph.2023.101351 (PMC9932564; doi:10.1016/j.ssmph.2023.101351)
Supplement: Multimedia component 1 [file mmc1.docx]

**Table S1:** STROBE-MR checklist of recommended items to address in reports of Mendelian randomization studies1 2

| **Item No.** | **Section** | **Checklist item** | **Page No.** | **Relevant text from manuscript** |
| --- | --- | --- | --- | --- |
| 1 | **TITLE and ABSTRACT** | Indicate Mendelian randomization (MR) as the study’s design in the title and/or the abstract if that is a main purpose of the study | 1 | “How Educational Attainment Modifies the Causal Relationship Between Adiposity and Cardiovascular Disease: A Mendelian Randomization Survival Analysis” |
|  | **INTRODUCTION** |  |  |  |
| 2 | **Background** | Explain the scientific background and rationale for the reported study. What is the exposure? Is a potential causal relationship between exposure and outcome plausible? Justify why MR is a helpful method to address the study question | 1-2 | The first paragraph explains the rationale of the study and exposure, the second paragraph explains why a causal relationship is plausible, and the third paragraph details why MR is useful. |
| 3 | **Objectives** | State specific objectives clearly, including pre-specified causal hypotheses (if any). State that MR is a method that, under specific assumptions, intends to estimate causal effects | 2 | Paragraph 3 details the goal of MR and paragraph 4 states the pre-specified hypothesis. |
|  | **METHODS** |  |  |  |
| 4 | **Study design and data sources** | Present key elements of the study design early in the article. Consider including a table listing sources of data for all phases of the study. For each data source contributing to the analysis, describe the following: |  |  |
|  | a) | Setting: Describe the study design and the underlying population, if possible. Describe the setting, locations, and relevant dates, including periods of recruitment, exposure, follow-up, and data collection, when available. | 5 | We describe the study design, population, setting, dates, etc. in the first paragraph of the methods section. |
|  | b) | Participants: Give the eligibility criteria, and the sources and methods of selection of participants. Report the sample size, and whether any power or sample size calculations were carried out prior to the main analysis | 5 | Paragraphs 2 and 3 of the Methods section and Figure 2 describe eligibility criteria for the study and the final sample size. |
|  | c) | Describe measurement, quality control and selection of genetic variants | 8-9 | Sections on selected genetic variants for BMI and WHRadjBMI and the appendix has a section on genetic variant quality that details the imputation process. |
|  | d) | For each exposure, outcome, and other relevant variables, describe methods of assessment and diagnostic criteria for diseases | 6 | In paragraphs labeled ‘incident cardiovascular disease’, ‘educational attainment’, and ‘anthropometry, we describe the methods for ascertaining the outcome, exposure, and stratifying variable. |
|  | e) | Provide details of ethics committee approval and participant informed consent, if relevant | N/A | Ethics Approval Section |
| 5 | **Assumptions** | Explicitly state the three core IV assumptions for the main analysis (relevance, independence and exclusion restriction) as well assumptions for any additional or sensitivity analysis | 10 | “Because they are randomly determined, these genetic variants can, in turn, serve as valid instrumental variables for adiposity subject to three assumptions: relevance, exchangeability, and no horizontal pleiotropy.” Second paragraph of Statistical Analyses section. |
| 6 | **Statistical methods: main analysis** | Describe statistical methods and statistics used |  |  |
|  | **a)** | Describe how quantitative variables were handled in the analyses (i.e., scale, units, model) | 6 | In paragraphs labeled ‘incident cardiovascular disease’, ‘educational attainment’, and ‘anthropometry, we describe the methods for ascertaining the outcome, exposure, and stratifying variable. |
|  | **b)** | Describe how genetic variants were handled in the analyses and, if applicable, how their weights were selected | 9; supplementary files | In Selected Genetic Variants section of the manuscript and in supplement. |
|  | **c)** | Describe the MR estimator (e.g. two-stage least squares, Wald ratio) and related statistics. Detail the included covariates and, in case of two-sample MR, whether the same covariate set was used for adjustment in the two samples | 11 | Detailed in Statistical Analyses section |
|  | **d)** | Explain how missing data were addressed | 10 | “We relied on complete case analysis due to the extremely low missingness in the UKB” |
|  | **e)** | If applicable, indicate how multiple testing was addressed | N/A | N/A |
| 7 | **Assessment of assumptions** | Describe any methods or prior knowledge used to assess the assumptions or justify their validity | 10-11;supplement | Described in Statistical Analyses section and in sensitivity analyses in the appendix |
| 8 | **Sensitivity analyses and additional analyses** | Describe any sensitivity analyses or additional analyses performed (e.g. comparison of effect estimates from different approaches, independent replication, bias analytic techniques, etc.) | 11-12;supplement | Sensitivity analyses detailed in Sensitivity Analyses section, replication of Neale Lab and GWAS results in Tables S2 and S3 and sensitivity analyses in supplement |
| 9 | **Software and pre-registration** |  |  |  |
|  | **a)** | Name statistical software and package, including version and settings used | 12 | All analyses are performed in R 4.1.3 |
|  | **b)** | State whether the study protocol and details were pre-registered (as well as when and where) | 3 | “All analyses and hypotheses were pre-specified and are available on Open Science Framework at <https://osf.io/38n9z/> |
|  | **RESULTS** |  |  |  |
| 10 | **Descriptive data** |  |  |  |
|  | **a)** | Report the numbers of individuals at each stage of included studies and reasons for exclusion. | 7 | Figure 2 on page 7 and accompanying text. |
|  | **b)** | Report summary statistics for phenotypic exposure, outcome, and other relevant variables | 12-13 | Tables 1 and 2 |
|  | **c)** | If the data sources include meta-analyses of previous studies, provide the assessments of heterogeneity across these studies | N/A | N/A |
|  | **d)** | For two-sample MR | N/A | N/A |
| 11 | **Main Results** |  |  |  |
|  | **a)** | Report the associations between genetic variant and exposure, and between genetic variant and outcome. | 12;14 | Table 3 for overall results and first stage partial F-statistic in first paragraph of results. |
|  | **b)** | Report MR estimates of the relationship between expousre and outcome, and the measures of uncertainty from the MR analysis. | 14 | Table 3 |
|  | **c)** | Consider translating estimates of relative risk into absolute risk for a meaningful time period | N/A | N/A. Already a measure of absolute hazard. |
|  | **d)** | Consider plots to visualize results. | 14-19 | Figures 3-5 |
| 12 | **Assessment of Assumptions** |  |  |  |
|  | **a)** | Report the assessment of the validity of assumptions. | 12; supplement | First pararaph of results section for relevance and supplemental file for sensitivity analyses on pleiotropy. |
|  | **b)** | Report any additional statistics. | N/A | N/A |
| 13 | **Sensitivity Analyses and Additional Analyses** |  |  |  |
|  | **a)** | Report any sensitivity analyses to assess the robustness of the main results to violations of the assumptions | 12; supplement | Weighted median model in Results section starting on page 12 and sensitivity analyses in the supplemental file |
|  | **b)** | Report results from other sensitivity analyses or additional analyses | N/A | N/A |
|  | **c)** | Report any assessment of direction of causal relationship. | N/A | N/A |
|  | **d)** | When relevant, report and compare with estimates from non-MR analyses. | 12 | Observational models are reported for each of the models in Table 3 and in Figures 3-5. |
|  | **e)** | Consider additional plots to visualize results | supplement | Conducted leave-one-out analyses with plots in supplement to assess influence of outlier genetic variants |
|  | **DISCUSSION** |  |  |  |
| 14 | **Key Results** | Summarize key results with reference to study objectives. | 20 | First two paragraphs of Discussion section |
| 15 | **Limitations** | Discuss limitations of the study, taking into account the validity of the IV assumptions, other sources of potential bias, and imprecision. Discuss both direction and magnitude of any potential bias and any efforts to address them. | 22-23 | Paragraphs two and three of Strengths & Limitations section |
| 16 | **Interpretation** |  |  |  |
|  | **a)** | Meaning: Give a cautious overall interpretation of results in the context of their limitations and in comparison with other studies. | 23 | Conclusion section |
|  | **b)** | Mechanism: Discuss underlying biological mechanisms that could drive a potential causal relationship between the investigated exposure and the outcome, and whether the gene-environment equivalence assumption is reasonable. Use causal language carefully, clarifying that IV estimates may provide causal effects only under certain assumptions. | 1-2 | Figure 1 and paragraph four of Introduction section |
|  | **c)** | Clinical relevance: Discuss whether the results have clinical or public policy relevance, and to what extent they inform effect sizes of possible interventions. | 23 | Fourth paragraph of Discussion section |
| 17 | **Generalizability** | Discuss the generalizability of the study results (a) to other populations, (b) across other exposure periods/timings, and (c) across other levels of exposure | 23 | Generalizability discussed in paragraph 2 of Strengths and Limitations section |
|  | **OTHER INFORMATION** |  |  |  |
| 18 | **Funding** | Describe sources of funding and the role of funders in the present study and, if applicable, sources of funding for the databases and original study or studies on which the present study is based | N/A | Funding section |
| 19 | **Data and data sharing** | Provide the data used to perform all analyses or report where and how the data can be accessed, and reference these sources in the article. Provide the statistical code needed to reproduce the results in the article, or report whether the code is publicly accessible and, if so, where. | 12 | First paragraph of Results section shows code and description of process for replication is available at <https://github.com/BobbySchell>. |
| 20 | **Conflicts of Interest** | All authors should declare all potential conflicts of interest | N/A | Acknowledgements section |

This checklist is copyrighted by the Equator Network under the Creative Commons Attribution 3.0 Unported (CC BY 3.0) license.

1. Skrivankova VW, Richmond RC, Woolf BAR, Yarmolinsky J, Davies NM, Swanson SA, et al. Strengthening the Reporting of Observational Studies in Epidemiology using Mendelian Randomization (STROBE-MR) Statement. JAMA. 2021;under review.
2. Skrivankova VW, Richmond RC, Woolf BAR, Davies NM, Swanson SA, VanderWeele TJ, et al. Strengthening the Reporting of Observational Studies in Epidemiology using Mendelian Randomisation (STROBE-MR): Explanation and Elaboration. BMJ. 2021;375:n2233.
