# Supplementary material for: Does educational attainment modify the causal relationship between adiposity and cardiovascular disease? A Mendelian randomization study
Source: SSM Popul Health. 2023 Jan 30;21:101351. doi: 10.1016/j.ssmph.2023.101351 (PMC9932564; doi:10.1016/j.ssmph.2023.101351)
Supplement: Multimedia component 2 [file mmc2.docx]

**Detailed Description of Inclusion Criteria in Fig 2**

First, we drop any subjects who were not genotyped (14,239 subjects). Because subjects of European ancestry are the largest group by far in the UK Biobank (comprising over 94% of the sample) and population stratification concerns prevent us from analyzing an ancestrally diverse sample, we then drop all individuals without European ancestry (78,620 subjects). Next, we remove individuals who withdrew consent to continue participating in data collection (96 subjects). We next drop all individuals related, defined as third degree relatives or closer, to at least one other person in the UK Biobank to avoid creating spurious associations caused by familial effects (131,927 subjects) (1,2).

We exclude individuals whose genetic data failed the standard inclusion quality control procedures created by the MRC Integrative Epidemiology Unit (731 subjects) (3). In short, this excludes individuals who have a mismatch between genetically inferred and reported sex, duplicates, and individuals who are outliers in terms of heterozygosity or missing rates. We exclude individuals with prevalent cardiovascular disease at baseline, defined as those who experienced a CVD event prior to their first adiposity measurement in the UKB (19,259 subjects) and those with missing adiposity measurement (1,278 subjects) or educational attainment status (1,979 subjects). The final sample size of the study was 254,281 participants with 27,511 cases of incident CVD.

**Detailed Description of Genetic Variant Imputation and Quality Control**

Because genetic variants are often co-inherited and inferring output from genotyping platforms can be difficult, imputation is a common technique used to allow a person’s available genetic data to predict any missing genotypes. Imputation will allow us to both keep individuals with incomplete genetic data, while also ensuring we can draw accurate inference from this population. The UK Biobank has developed their own imputation methods, detailed in the documentation at <https://biobank.ndph.ox.ac.uk/showcase/showcase/docs/impute_ukb_v1.pdf>. To assess the quality of the UKB’s imputation for our variants, we rely on INFO score, a measure of genetic variants’ imputation quality scaled between 0 and 1, with a 1 denoting perfect imputation. Every genetic variant in this analysis had an INFO score higher than 0.95, which signifies the high level of imputation accuracy in this analysis. For individuals with imputed SNPs, we perform hard-call genotyping, which assigns variants to the most likely allele count between 0, 1, and 2 based on an individual’s related genetic information.

We next checked for the existence of multi-allelic or palindromic SNPs. In brief, a multi-allelic SNP is one with more than the conventional two alleles, which would make applying any of our estimators impossible. Palindromic SNPs are those with the same letters on the forward and reverse strands, which makes identifying the effect allele challenging, particularly if the effect allele frequency is roughly 0.5. We identified no multi-allelic but two palindromic SNPs:rs1558902 (16:52,361,075) and rs9641123 (7:93,035,668) for BMI. We reran these models without the two above SNPs, which did not affect the results. No such variants existed for WHRadjBMI.

**Figure S1: Follow-up time by event type for body mass index and waist-to-hip ratio adjusted for body mass index**

*BMI*


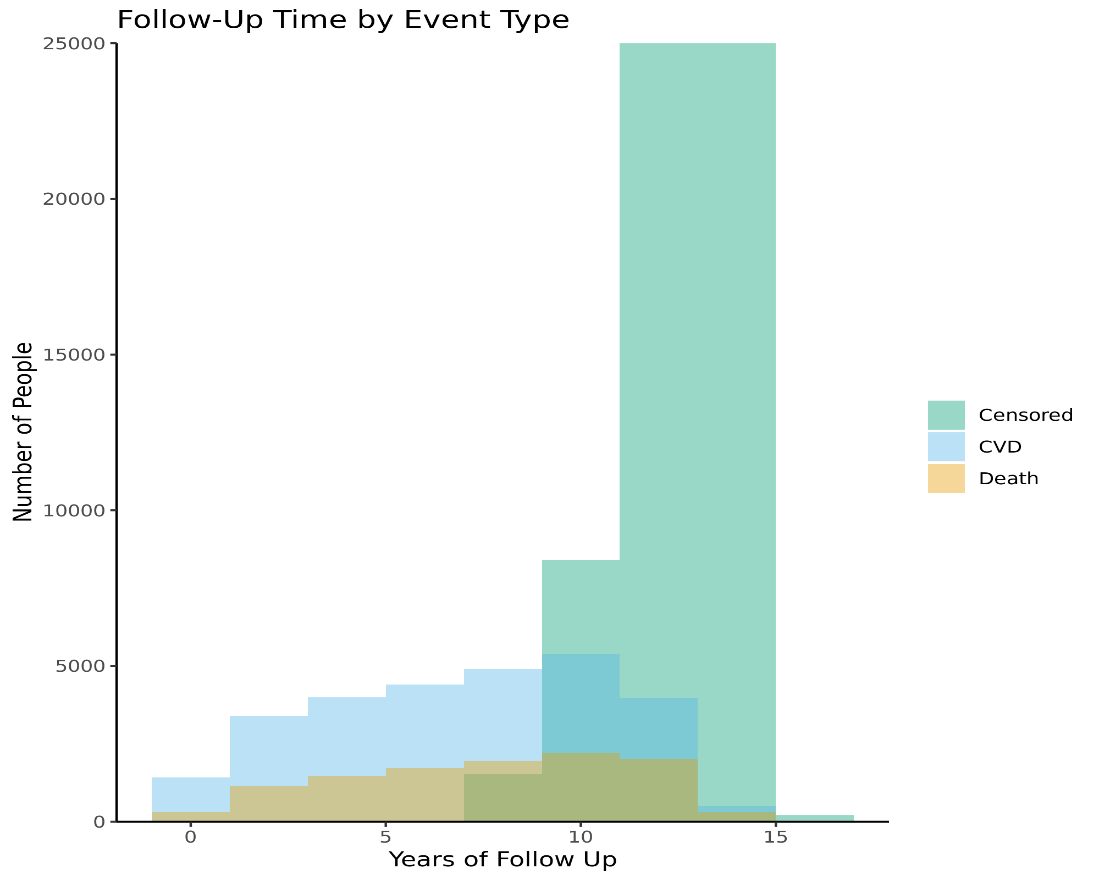


*WHRadjBMI*


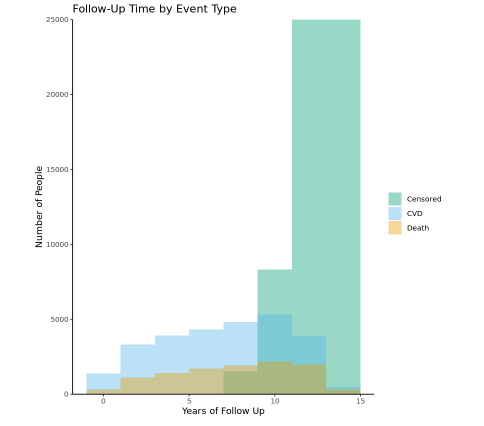


**Figure S2: Kaplan-Meier survival probability for body mass index and waist-to-hip ratio adjusted for body mass index**


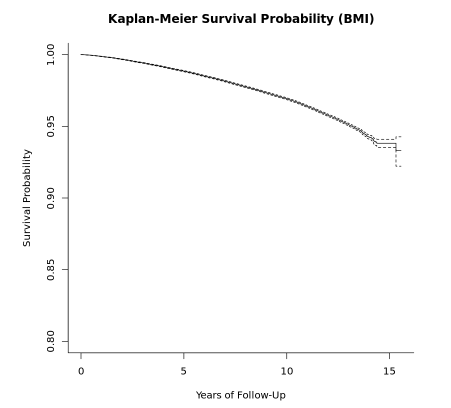

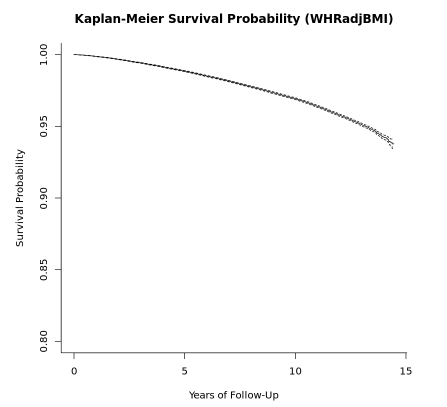


**Figure S3: Cumulative incidence functions for body mass index and waist-to-hip ratio adjusted for body mass index**


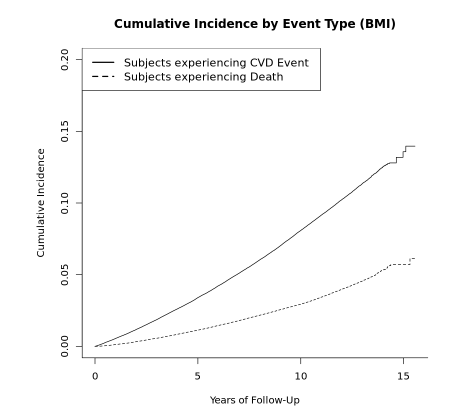


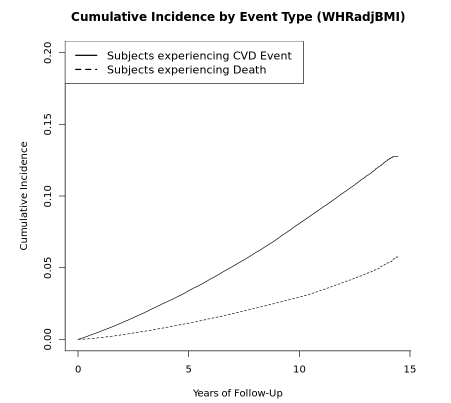


**Sensitivity Analyses**

The simplest method to minimize the potential role of horizontal pleiotropy in the analysis is a “leave one out” method, where the model is re-estimated excluding each SNP separately (4). This analysis should demonstrate whether any outlier SNPs have a disproportionate impact on the effect estimate. If each of these iterations provides directionally consistent results, it decreases the likelihood that horizontal pleiotropy plays a large role in driving the existence of a causal effect because the different SNP subsets would likely not suffer from the exact same source of bias.

A more direct way of assessing the causal effect of adiposity on CVD would involve including only SNPs with an established mechanistic link to adiposity that makes their association more plausible (5). BMI is determined by a host of mechanisms not directly related to adiposity, we are only able to perform this sensitivity analysis for WHRadjBMI. Out of the original set of 49 SNPs in WHRadjBMI, only 9 are associated with gene expression in either subcutaneous or omental adipose tissue. Because there were only 9 variants associated with WHRadjBMI that had a plausible functional component identified, we tested the strength of this instrument. The combined effect of the genetic variants produced an F-statistic under 10, suggesting the estimator would suffer from weak instrument bias. As a result, we did not perform this sensitivity analysis. As a result, we only present the leave-one-out analyses below.

| **Figure S4: Leave-one-out analyses for waist-to-hip ratio adjusted for body mass index (Female)** | |
| --- | --- |
| 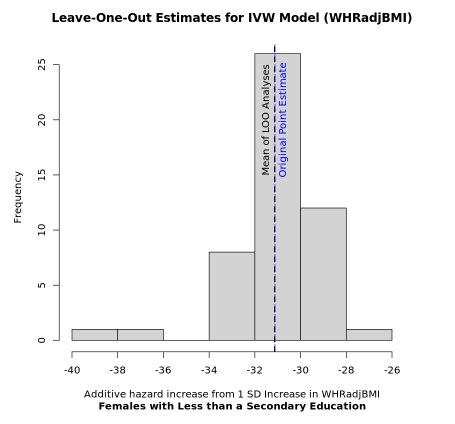 | 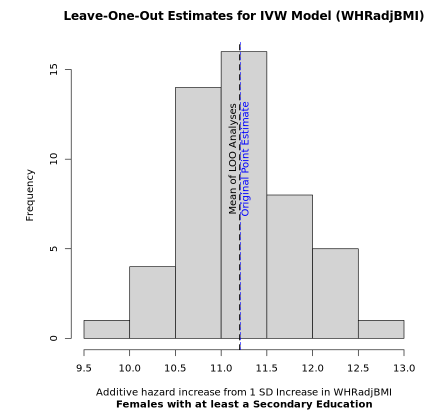 |
| 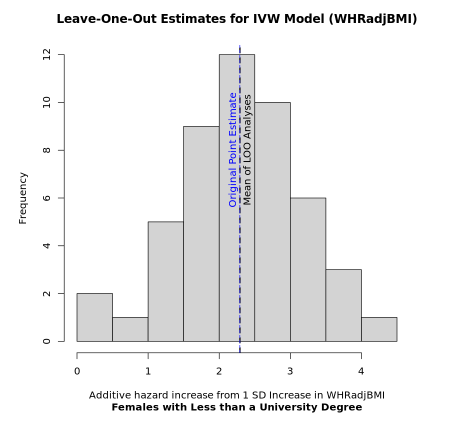 | 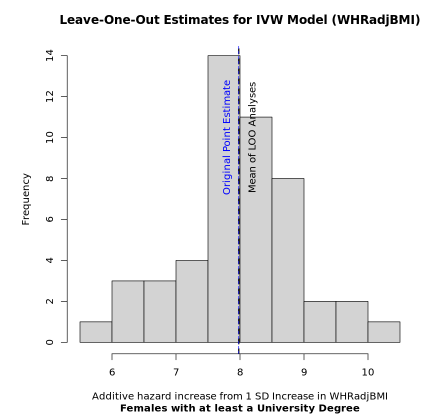 |

The above figs plot the distribution of point estimates across the iterations of the leave-one-out (LOO) analyses. The LOO analysis for the effect of WHRadjBMI on incident CVD for females suggests that no one genetic variant appeared to have an outsized influence on the results except in the case of females with less than a secondary education. However, because this effect was already so imprecisely estimated, it is unclear how much these genetic variants actually impact the observed results.

| **Figure S5: Leave-one-out analyses for waist-to-hip ratio adjusted for body mass index (Male)** | |
| --- | --- |
| 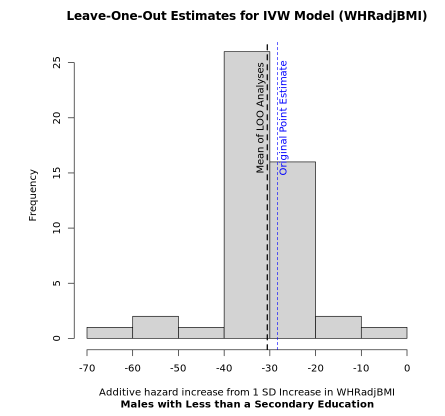 | 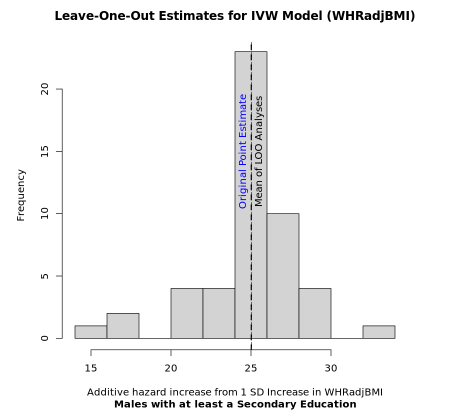 |
| 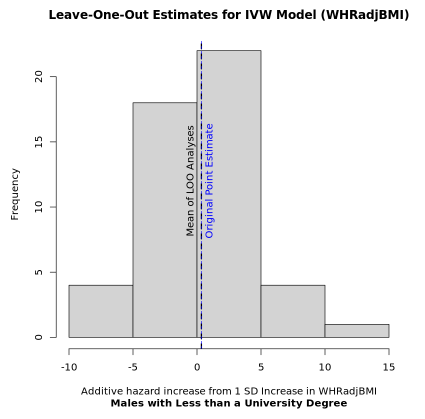 | 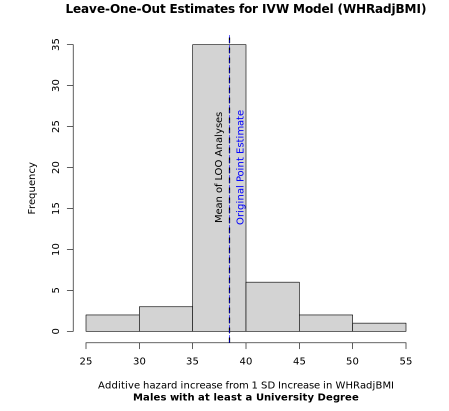 |

The LOO analysis for the effect of WHRadjBMI on incident CVD for males suggests that no one genetic variant appeared to have an outsized influence on the results except in the case of females with less than a secondary education. There were some notable outliers for males with at least a secondary education and a wide range for males with at least a university degree.

| **Figure S6: Leave-one-out analyses for body mass index** | |
| --- | --- |
| 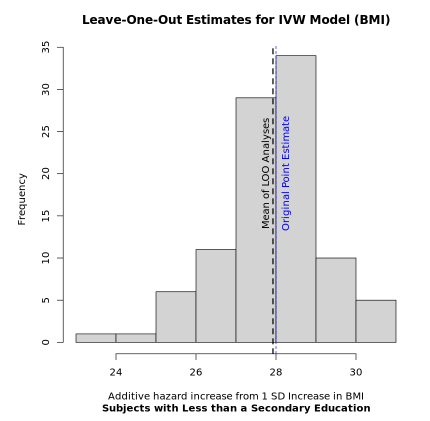 | 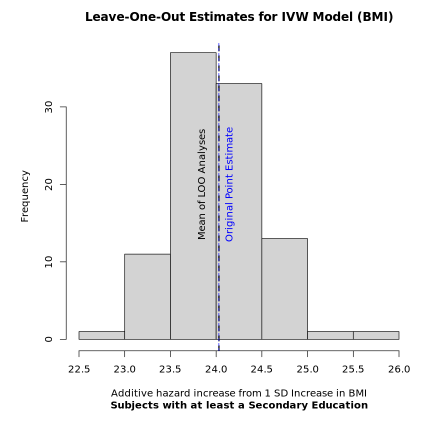 |
| 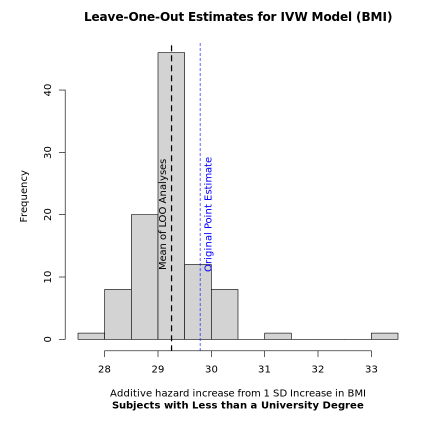 | 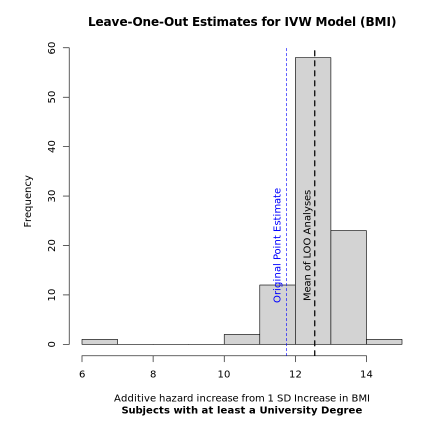 |

The LOO analysis for the effect of BMI on incident CVD, which produced far more precise estimates than WHRadjBMI, unsurprisingly demonstrates more consistency in results across genetic variants. There are notable outliers for subjects with less than and at least a university degree; however, these were of much smaller magnitude than the outliers in the WHRadjBMI analysis.

*Functional SNPs ONLY (WHRadjBMI)*

Because there were only 9 variants associated with WHRadjBMI that had a plausible functional component identified, we tested the strength of this instrument. The combined effect of the genetic variants produced an F-statistic under 10, suggesting the estimator would suffer from weak instrument bias. As a result, we did not perform this sensitivity analysis.

**Table S2: Association between adiposity and incident cardiovascular disease by household income and model choice**

|  |  | **Body Mass Index** | | **WHRadjBMI**  **(Male)** | | **WHRadjBMI**  **(Female)** | |
| --- | --- | --- | --- | --- | --- | --- | --- |
|  |  | **Additive Hazard** | **95% Confidence Interval** | **Additive Hazard** | **95% Confidence Interval** | **Additive Hazard** | **95% Confidence Interval** |
| HH Income Under £18,000 | Associational | 29.45 | (26.08, 32.83) | 33.00 | (23.00, 43.00) | 13.40 | (8.54, 18.30) |
|  | IVW | 25.04 | (5.25, 44.84) | -93.80 | (-195.20, 7.60) | 9.61 | (-14.69, 33.92) |
|  | Weighted Median | 13.79 | (-18.90, 46.48) | -70.25 | (-216.15, 75.65) | 13.16 | (-22.26, 48.57) |
| HH Income at least £18,000 | Associational | 19.46 | (18.10, 20.82) | 17.70 | (14.20, 21.20) | 3.94 | (2.08, 5.80) |
|  | IVW | 25.28 | (15.66, 34.85) | 29.83 | (-11.07, 70.72) | 4.99 | (-4.55, 14.53) |
|  | Weighted Median | 27.76 | (12.94, 42.59) | 16.76 | (-37.74, 71.26) | 3.46 | (-10.55, 17.46) |

**Figure S7: Associational relationship between adiposity and incident cardiovascular disease**

*BMI*


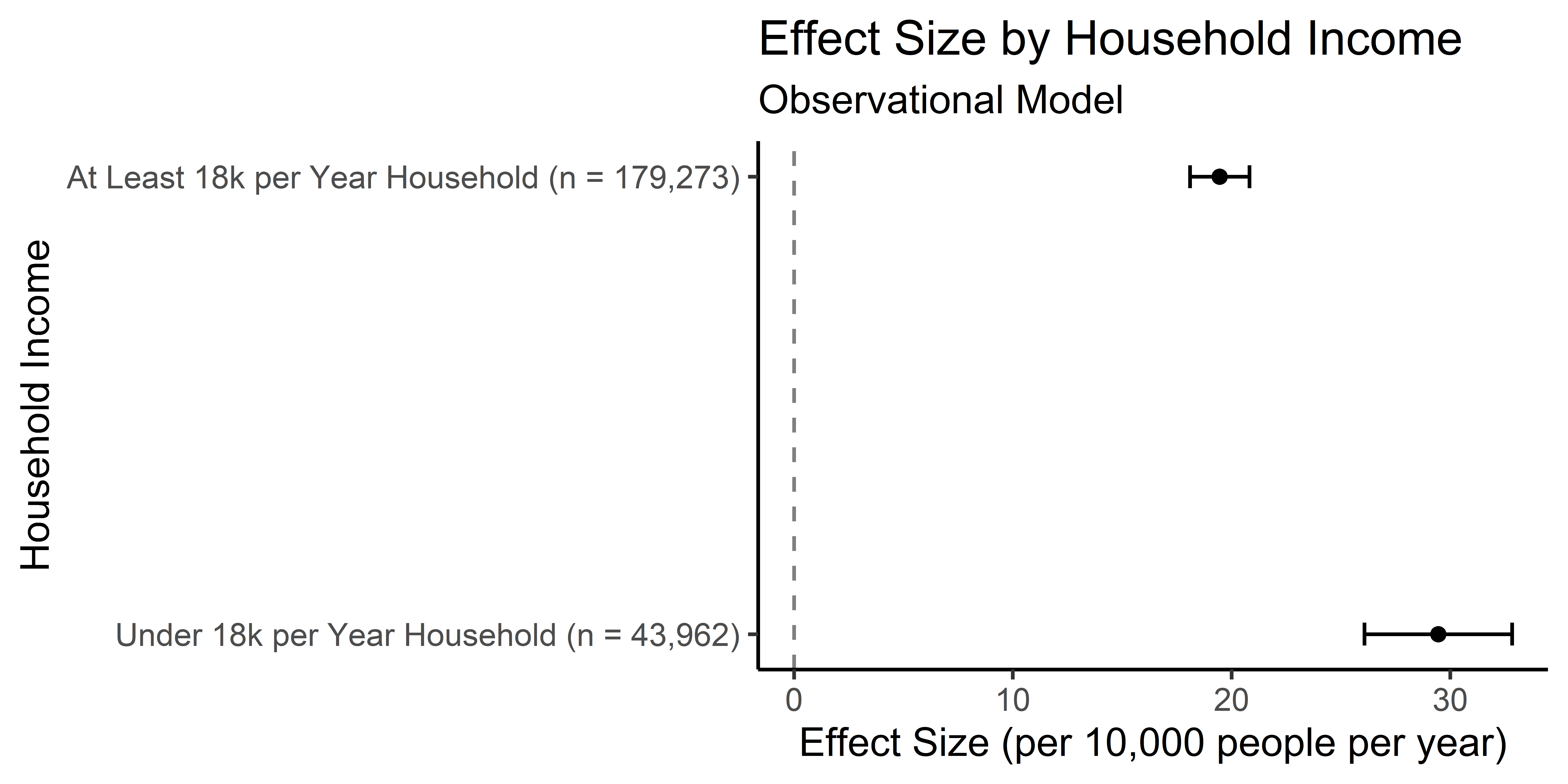


*WHRadjBMI (Male)*


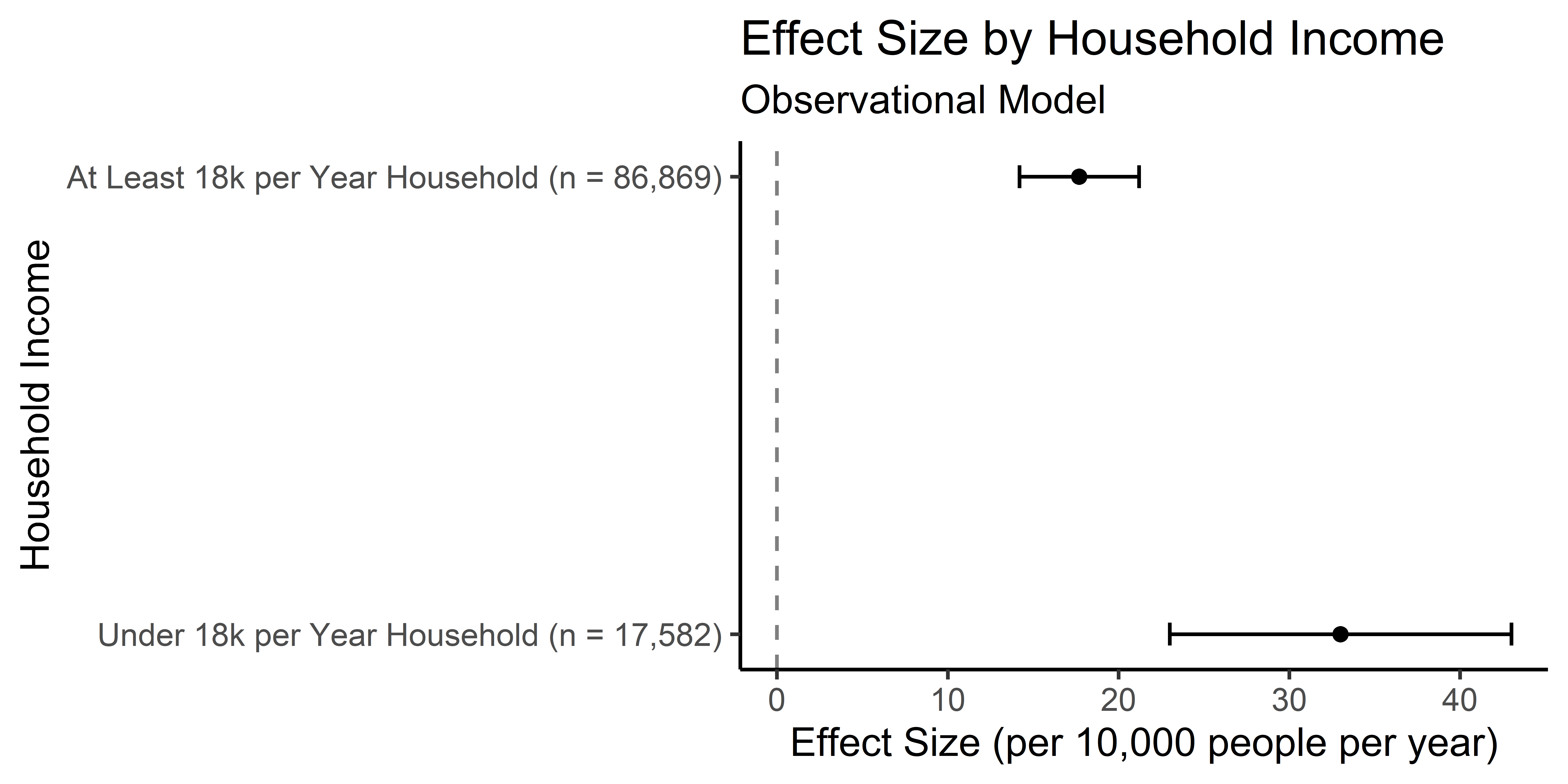


*WHRadjBMI (Female)*


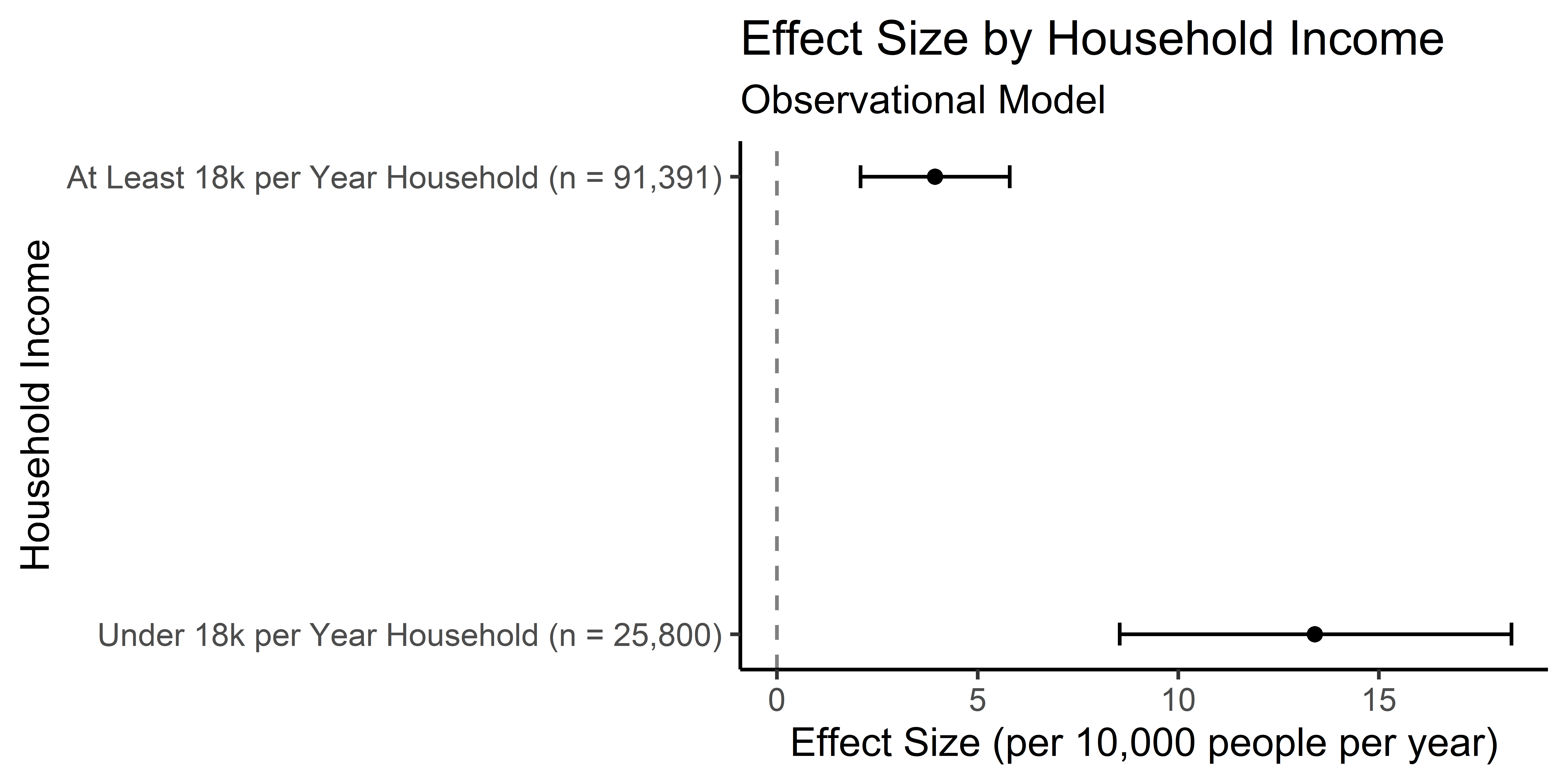


**Figure S8: IVW association between adiposity and incident cardiovascular disease**

*BMI*


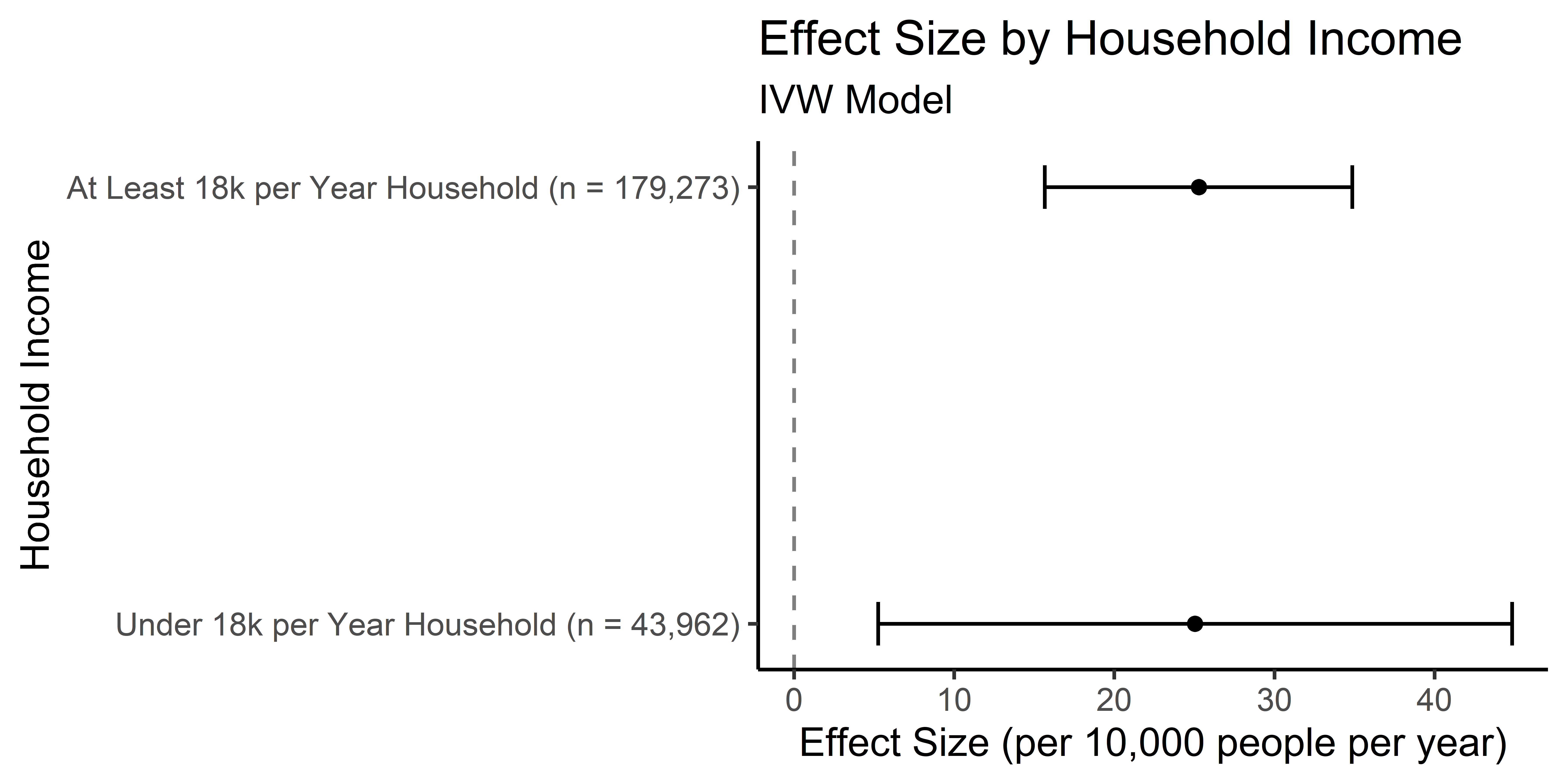


*WHRadjBMI (Male)*


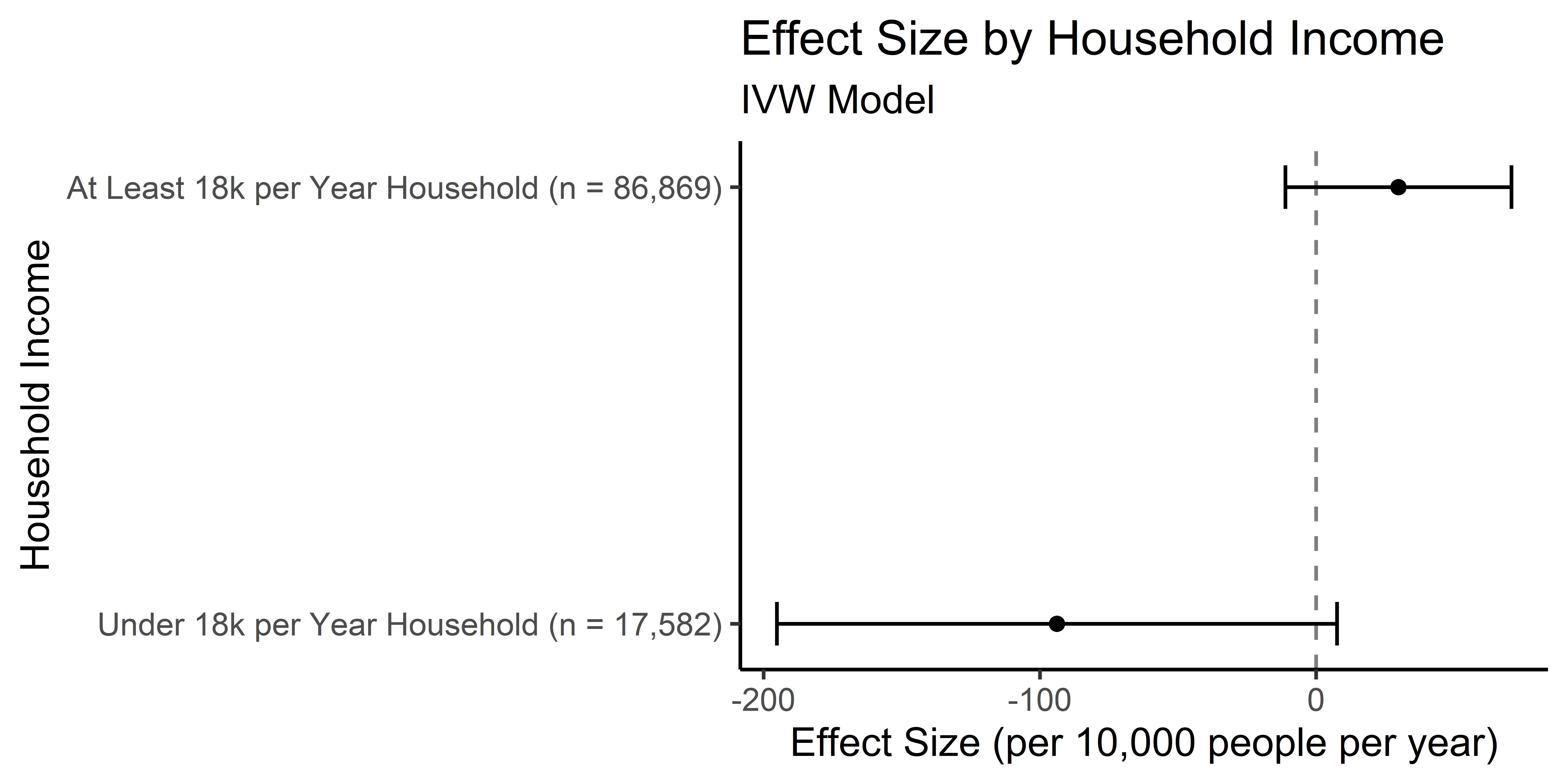


*WHRadjBMI (Female)*


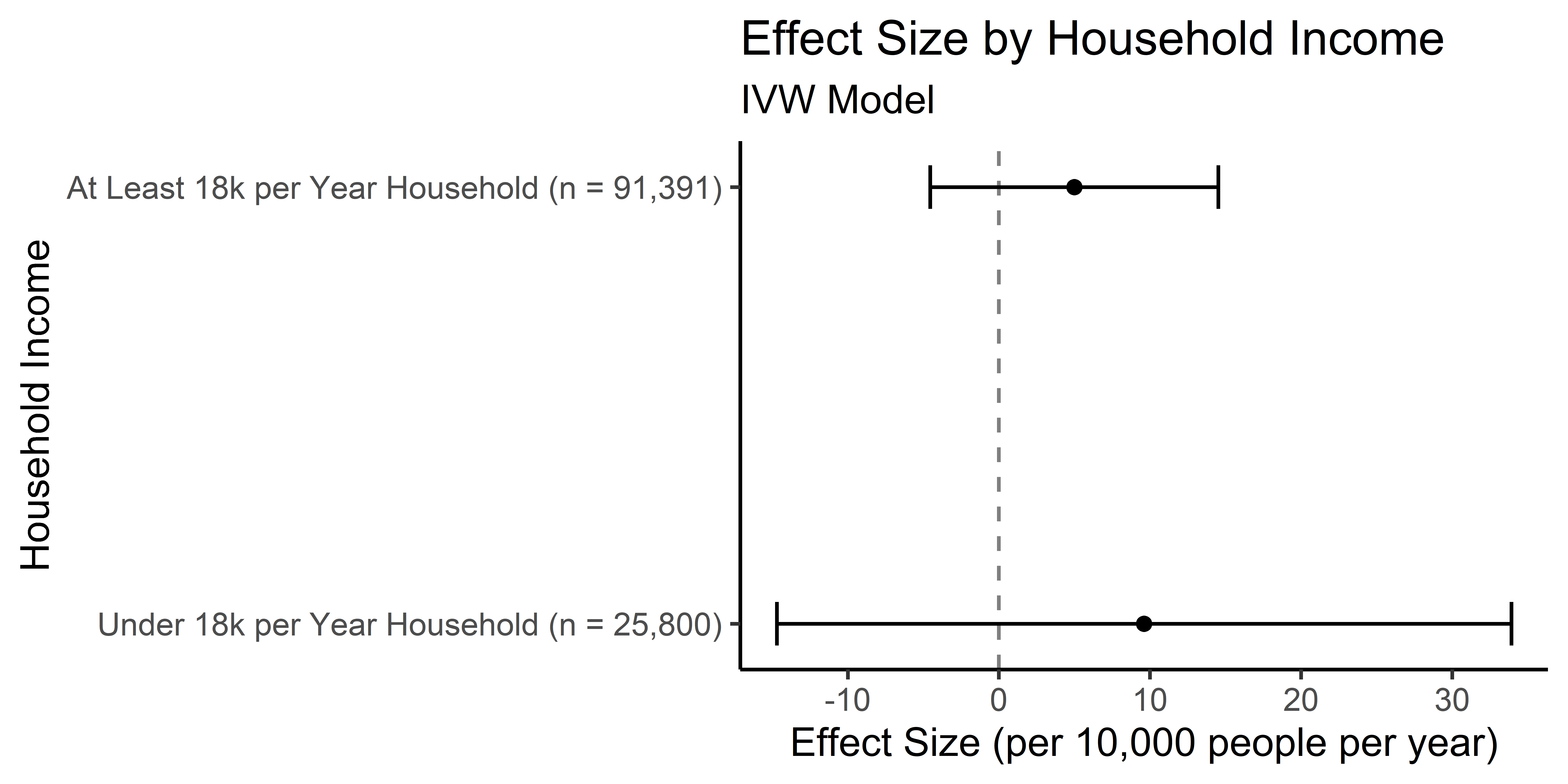


**Figure S9: Weighted median association between adiposity and incident cardiovascular disease**

*BMI*


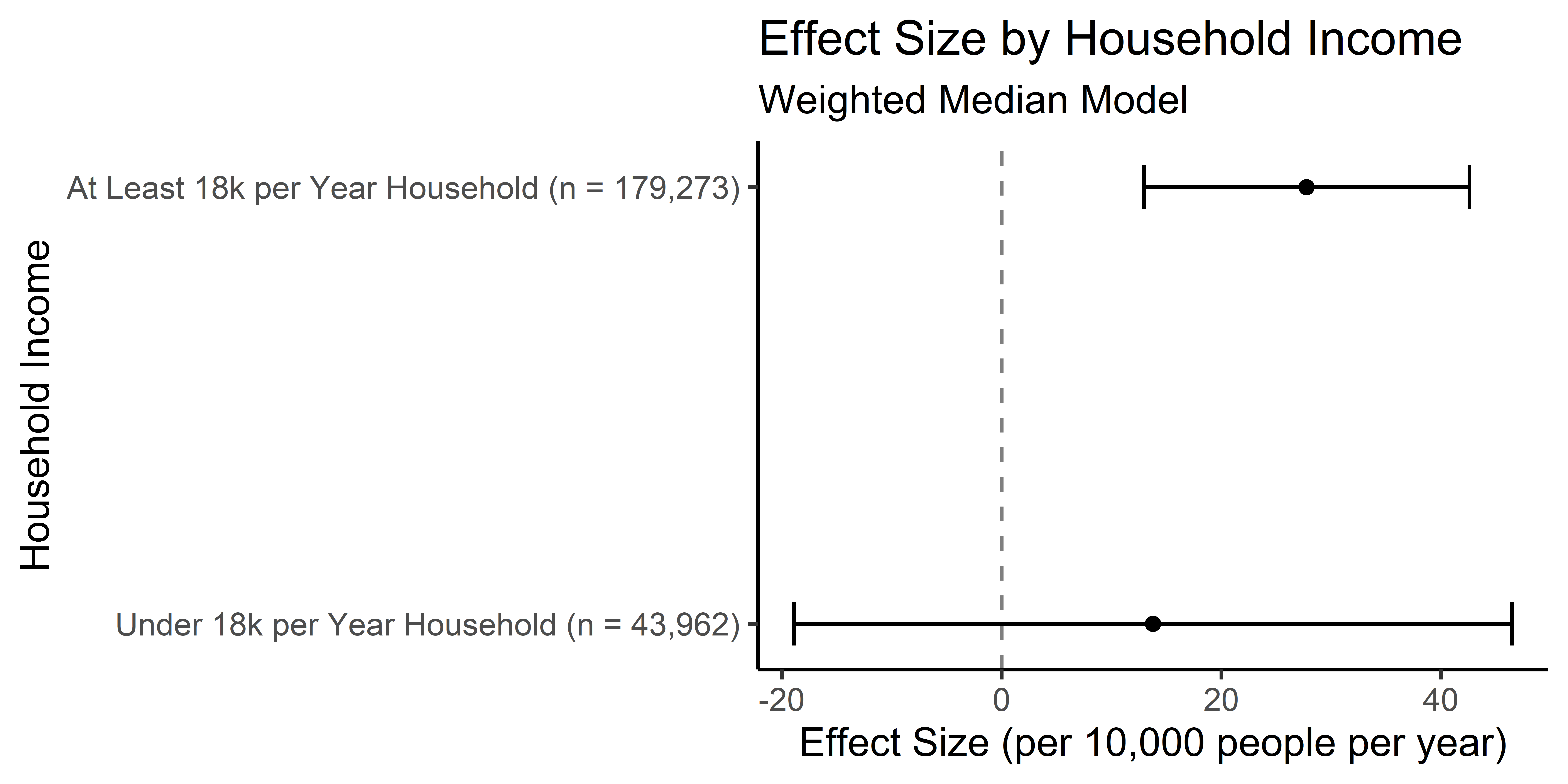


*WHRadjBMI (Male)*


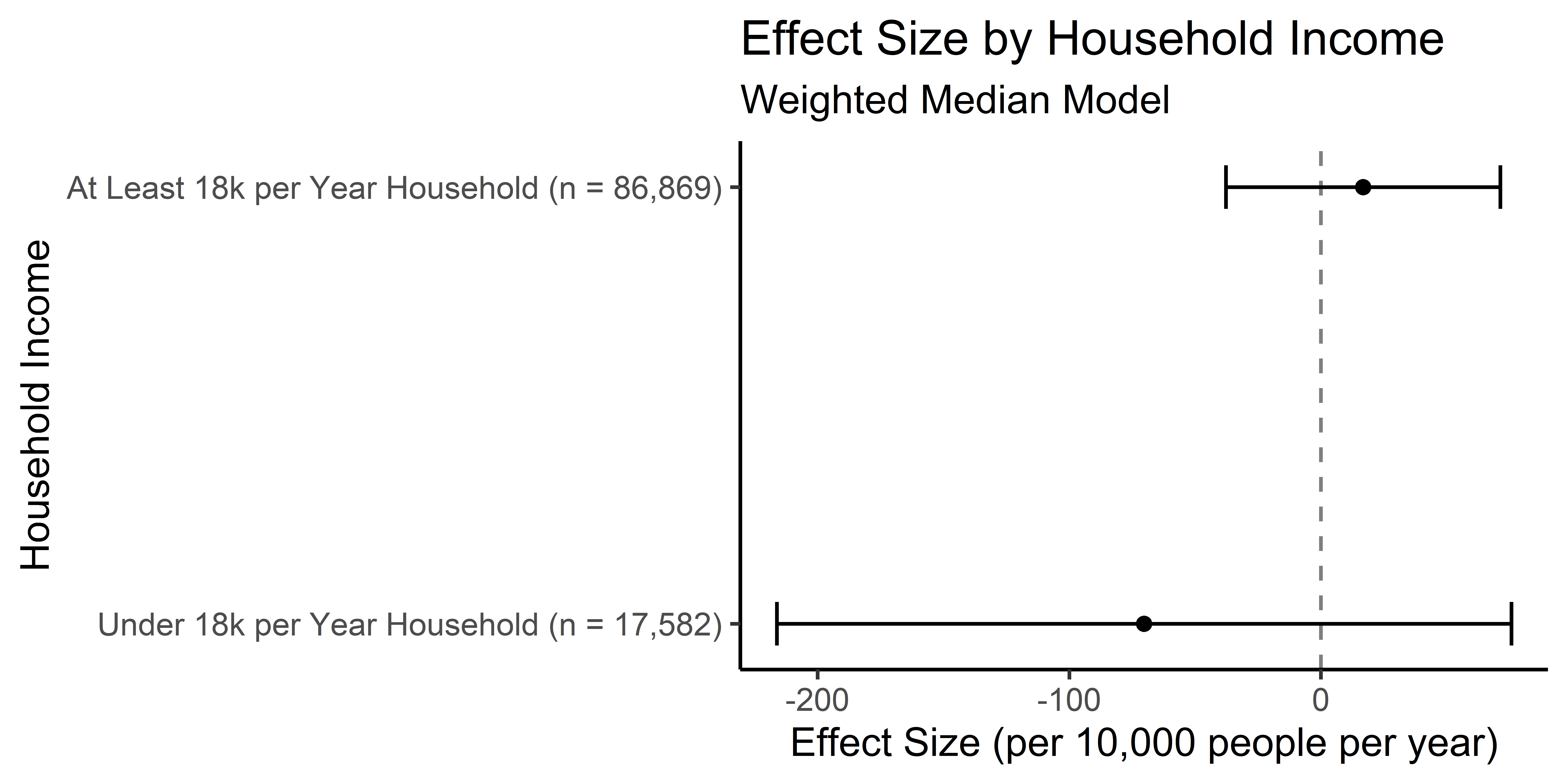


*WHRadjBMI (Female)*


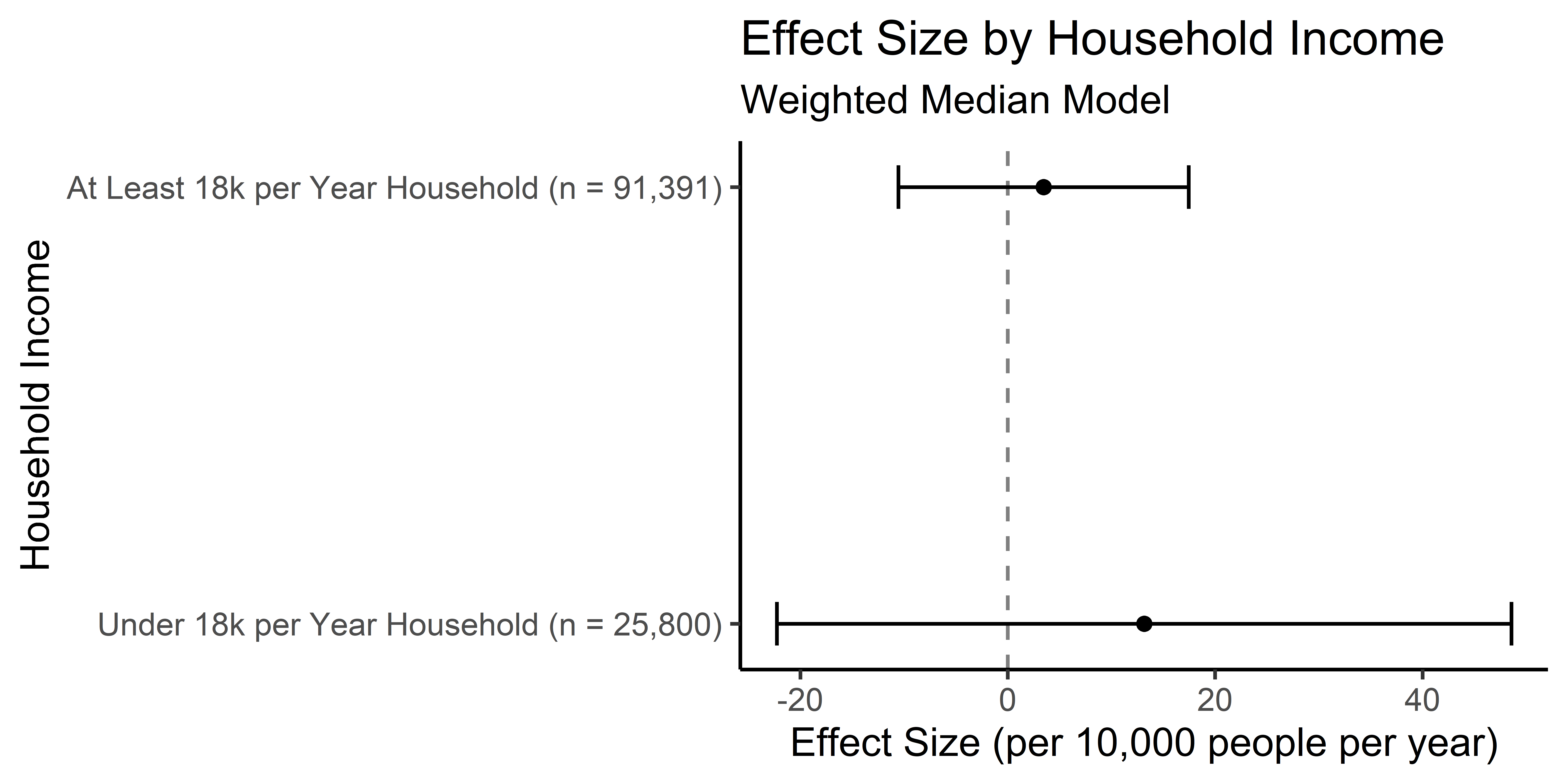


References

1. Brumpton B, Sanderson E, Heilbron K, Hartwig FP, Harrison S, Vie GÅ, et al. Avoiding dynastic, assortative mating, and population stratification biases in Mendelian randomization through within-family analyses. Nat Commun 2020 111 [Internet]. 2020 Jul 14 [cited 2022 May 6];11(1):1–13. Available from: https://www.nature.com/articles/s41467-020-17117-4

2. Bycroft C, Freeman C, Petkova D, Band G, Elliott LT, Sharp K, et al. The UK Biobank resource with deep phenotyping and genomic data. Nature. 2018 Oct 10;562(7726):203–9.

3. Mitchell R, Hemani G, Dudding T, Corbin L, Harrison S, Paternoster L. UK Biobank Genetic Data: MRC-IEU Quality Control, version 2. [Internet]. 2019. Available from: https://doi.org/10.5523/bris.1ovaau5sxunp2cv8rcy88688v

4. Hemani G, Bowden J, Davey Smith G. Evaluating the potential role of pleiotropy in Mendelian randomization studies. Hum Mol Genet [Internet]. 2018 Aug 1 [cited 2022 Mar 24];27(R2):R195–208. Available from: https://academic.oup.com/hmg/article/27/R2/R195/4996734

5. S W, JR M, LD K, ER M, PK C, SC C, et al. Diabetic Phenotypes and Late-Life Dementia Risk: A Mechanism-specific Mendelian Randomization Study. Alzheimer Dis Assoc Disord [Internet]. 2016 [cited 2021 Nov 5];30(1):15–20. Available from: https://pubmed.ncbi.nlm.nih.gov/26650880/
